# New Taxane Diterpenoids from *Taxus yunnanensis*

**DOI:** 10.1007/s13659-014-0003-9

**Published:** 2014-02-19

**Authors:** Ping Hai, Shi-Zhen Wen, Yan Li, Yuan Gao, Xian-Jun Jiang, Fei Wang

**Affiliations:** 1 BioBioPha Co., Ltd., Kunming, 650201 China; 2 State Key Laboratory of Phytochemistry and Plant Resources in West China, Kunming Institute of Botany, Chinese Academy of Sciences, Kunming, 650201 China

**Keywords:** *Taxus yunnanensis*, Taxane, Baccatin, Cytotoxicity

## Abstract

Three hitherto unknown taxane diterpenoids, namely baccatin VIII (**1**), baccatin IX (**2**), and baccatin X (**3**), along with 10 known analogues were isolated from an ethanolic extract of the twigs and leaves of *Taxus yunnanensis*. The new structures were characterized based on extensive spectroscopic analysis. Compounds **1** and **2** were tested for their in vitro cytotoxicity against five human tumor cell lines, and **1** exhibited inhibitory effects on HL-60 and MCF-7, with IC_50_ values of 3.44 and 9.67 μM, respectively.

**Electronic supplementary material:**

The online version of this article (doi:10.1007/s13659-014-0003-9) contains supplementary material, which is available to authorized users.

## Introduction

Plants of the genus *Taxus* (Taxaceae) are large, ornamental evergreen shrubs or trees, most of which are distributed in the Northern hemisphere and have attracted much attention owing to natural taxane diterpenoid Taxol, an antitumor agent for treating the sufferers of ovarian, breast, and other carcinomas [1, 2]. Several groups conducted a series of phytochemical research work on the *Taxus* species, and more than 550 taxane diterpenoids have been isolated from this genus [2–5]. *Taxus yunnanensis*, a slow-growing tree commonly known as “Yunnan Hongdoushan” and grown mainly in Yunnan Province of China, is considered as a promising source of taxane diterpenes [6]. With the aim of isolating more Taxol derivatives with biological activities, we investigated the constituents of the twigs and leaves of *T. yunnanensis* once again, which led to the isolation of three new taxane diterpenoids baccatins VIII, IX, and X (**1**–**3**), together with 10 known analogues, baccatin III (**4**) [7], 10-deacetylbaccatin III (**5**) [7], 19-hydroxybaccatin III (**6**) [8], 14*β*-benzoyloxy-2-deacetylbaccatin VI (**7**) [9], baccatin IV (**8**) [10], 1-hydroxybaccatin I (**9**) [7], 13-*O*-deacetyltaxumairol Z (**10**) [11], taxayunnansin A (**11**) [12], 2-deacetoxytaxinine B (**12**) [13], and taxinine B (**13**) [14]. This paper reports on the isolation and structure determination of the new taxanes and their cytotoxicity.

## Results and Discussion

Compound **1** was obtained as a white amorphous powder. The HRESIMS data (*m*/*z* 669.2513 [M + Na]^+^) indicated the molecular formula C_33_H_42_O_13_, requiring 13 degrees of unsaturation. The IR absorption bands at 3433 and 1726 cm^−1^ suggested the presence of hydroxy and carbonyl functionalities, respectively. The ^1^H NMR spectrum of **1** (Table 1) displayed signals of four tertiary methyls (*δ*_H_ 1.19, 1.65, 1.77, and 1.88), three acetyl methyls (*δ*_H_ 2.10, 2.23, and 2.34), seven oxygenated methines (*δ*_H_ 4.03, 4.37, 4.51, 4.92, 5.87, 6.00, and 6.17), a benzoyl group (*δ*_H_ 8.07 × 2, 7.48 × 2, and 7.61), and an oxetane ring (*δ*_H_ 4.21 and 4.17, each 1H, d, *J* = 8.2 Hz). Beside the resonances for three acetyl groups (*δ*_C_ 171.2, 22.9; 171.9, 21.3; 172.3, 21.2) and a benzoyl group (*δ*_C_ 167.1, 130.6, 129.4 × 2, 130.8 × 2, and 134.3), 20 carbon signals, including an oxygenated methylene carbon (*δ*_C_ 77.3), seven oxygenated methines (*δ*_C_ 70.5, 73.8, 74.2, 74.5, 77.6, 79.8, 85.2), two oxygenated quaternary carbons (*δ*_C_ 76.8 and 82.8), and a tetrasubstituted double bond (*δ*_C_ 137.3 and 137.7) were displayed in the ^13^C NMR and DEPT spectra of **1** (Table 1). Comparison of the NMR data with those of 9-dihydro-13-*O*-acetylbaccatin III [15] revealed that **1** was a taxane diterpenoid very similar to it. The only difference between them was that a methylene (*δ*_C_ 36.1, C-14) in 9-dihydro-13-*O*-acetylbaccatin III was replaced by a hydroxylated methine (*δ*_C_ 70.5) in **1**. This was further confirmed by the HMBC cross-peaks of H-14 (*δ*_H_ 4.03, d, *J* = 6.7 Hz) with C-1 (*δ*_C_ 76.8, s), C-13 (*δ*_C_ 79.8, d), and C-15 (*δ*_C_ 43.8, s).

**Table 1:** NMR data for compounds **1**–**3**

No.	1^a^		2^b^		3^a^	
	*δ*_H_ (*J* in Hz)	*δ*_C_	*δ*_H_ (*J* in Hz)	*δ*_C_	*δ*_H_ (*J* in Hz)	*δ*_C_
1		76.8 (s)		75.6 (s)		79.0 (s)
2	5.87 (d, 6.1)	73.8 (d)	5.65 (d, 6.0)	72.7 (d)	4.02 (d, 6.0)	74.2 (d)
3	3.01 (d, 6.1)	47.1 (d)	2.89 (d, 6.0)	46.0 (d)	2.86 (d, 6.0)	48.7 (d)
4		82.8 (s)		80.8 (s)		83.9 (s)
5	4.92 (br. d, 9.0)	85.2 (d)	4.82 (br. d, 9.2)	83.3 (d)	5.00 (dd, 9.3, 1.3)	85.2 (d)
6*α*	2.46 (ddd, 15.0, 9.0, 8.0)	38.2 (t)	2.28 (ddd, 14.8, 9.2, 7.2)	37.7 (t)	2.47 (ddd, 14.7, 9.3, 7.5)	38.8 (t)
6*β*	1.84 (ddd, 15.0, 10.0, 0.9)		1.63 (ddd, 14.8, 10.3, 0.8)		1.86 (ddd, 14.7, 10.0, 1.3)	
7	4.37 (dd, 10.0, 8.0)	74.5 (d)	4.21 (overlap)	73.2 (d)	4.41 (dd, 10.0, 7.5)	75.4 (d)
8		45.4 (s)		44.0 (s)		45.6 (s)
9	4.51 (d, 11.0)	77.6 (d)	4.25 (overlap)	76.1 (d)	4.29 (d, 11.0)	78.3 (d)
10	6.17 (d, 11.0)	74.2 (d)	6.01 (d, 10.8)	73.3 (d)	6.21 (d, 11.0)	75.0 (d)
11		137.7 (s)		134.3 (s)		136.4 (s)
12		137.3 (s)		141.1 (s)		142.3 (s)
13	6.00 (dq, 6.7, 0.9)	79.8 (d)	4.38 (br. dd, 5.9, 5.6)	75.5 (d)	4.94 (overlap)	74.3 (d)
14	4.03 (d, 6.7)	70.5 (d)	3.80 (dd, 6.6, 5.9)	72.4 (d)	5.05 (d, 6.1)	79.1 (d)
15		43.8 (s)		42.2 (s)		43.8 (s)
16	1.19 (s)	28.7 (q)	0.96 (s)	28.4 (q)	1.29 (s)	28.9 (q)
17	1.65 (s)	24.4 (q)	1.46 (s)	23.9 (q)	1.52 (s)	23.8 (q)
18	1.88 (d, 0.9)	14.9 (q)	1.90 (s)	15.2 (q)	2.06 (d, 1.3)	15.6 (q)
19	1.77 (s)	13.0 (q)	1.60 (s)	12.5 (q)	1.77 (s)	13.1 (q)
20*α*	4.21 (d, 8.2)	77.3 (t)	3.96 (br. s)	75.4 (t)	4.70 (d, 8.8)	79.1 (t)
20*β*	4.17 (d, 8.2)				4.60 (d, 8.8)	
1′		167.1 (s)		165.4 (s)		169.3 (s)
2′		130.6 (s)		129.9 (s)		131.4 (s)
3′, 7′	8.07 (br. d, 7.8)	130.8 (d)	8.02 (br. d, 7.8)	129.9 (d)	8.09 (br. d, 8.3)	130.9 (d)
4′, 6′	7.48 (dd, 7.8, 7.6)	129.4 (d)	7.51 (dd, 7.8, 7.6)	128.8 (d)	7.49 (dd, 8.3, 7.6)	129.5 (d)
5′	7.61 (br. t, 7.6)	134.3 (d)	7.64 (br. t, 7.6)	133.4 (d)	7.62 (br. t, 7.6)	134.4 (d)
4-CO*CH*_*3*_	2.34 (s)	22.9 (q)	2.18 (s)	22.7 (q)	2.08 (s)	22.9 (q)
4-*CO*CH_3_		171.2 (s)		169.7 (s)		172.0 (s)
10-CO*CH*_*3*_	2.10 (s)	21.3 (q)	2.01 (s)	21.2 (q)	2.08 (s)	21.3 (q)
10-*CO*CH_3_		171.9 (s)		169.9 (s)		172.2 (s)
13-CO*CH*_*3*_	2.23 (s)	21.2 (q)				
13-*CO*CH_3_		172.3 (s)				
1-OH			4.40 (s)			
7-OH			6.30 (d, 3.5)			
9-OH			6.31 (d, 3.0)			
13-OH			5.46 (d, 5.6)			
14-OH			6.74 (d, 6.6)			

^a^ Measured in CD_3_OD

^b^ Measured in DMSO-*d*_6_

**Fig. 1:**
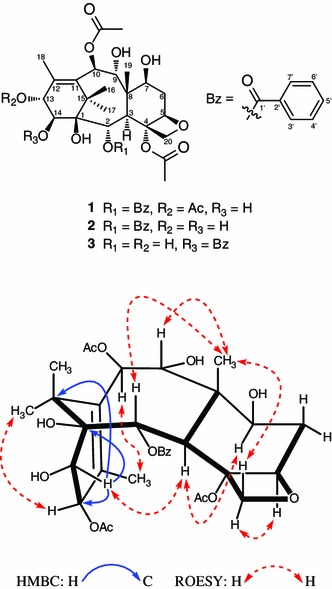
Key HMBC and ROESY correlations of **1**

The relative configuration of **1** was deduced from the ROESY experiment (Fig. 1). The significant correlations of H-14/H-3 and H-3/H-7 were observed, indicating *α*-orientation of these protons. Similarly, the correlations of H-2/Me-19, Me-19/H-9, and Me-19/H-20*β* were observed, revealing that these protons were on the other face of the molecule. *β*-Orientation of H-13 was suggested by the strong correlation between H-13 and Me-16, while *α*-orientation of H-10 and H-5 was deduced by the correlations of H-10/Me-18 and H-5/H-20*α*, respectively. In addition, the hydroxy group attached to the bridgehead carbon was assigned as *β*-oriented, based on ring junction inferred by the correlations of H-13/Me-16 and H-14/H-3. Thus, the structure of **1** was unambiguously identified as shown, and given the trivial name baccatin VIII.

Compound **2** was isolated as a white powder with the molecular formula C_31_H_40_O_12_, as determined by HREIMS: *m*/*z* 604.2515 [M]^+^ (calcd for C_31_H_40_O_12_, 604.2520). Analysis of its NMR data (Table 1) indicated that **2** closely resembled **1** except for the absence of signals for an acetyl and a dramatic upfield shift of H-13 from *δ*_H_ 6.00 in **1** to *δ*_H_ 4.38 in **2**. This indicated an absence of the acetyl at C-13, as established by the HMBC correlations of 13-OH (*δ*_H_ 5.46, d, *J* = 5.6 Hz) with C-12 (*δ*_C_ 141.1), C-13 (*δ*_C_ 75.5), and C-14 (*δ*_C_ 72.4). The relative configuration of 13-OH was assigned as *α* on the basis of the ROESY correlation of H-13 with Me-16. The stereochemistry of other chiral centers was in accordance with that of **1**, by the ROESY correlations of H-2/Me-19, H-3/H-7, H-3/H-5, H-3/H-14, H-10/Me-18, and Me-19/H-20*β*. The structure of **2** was therefore characterized as shown and named baccatin IX.

Compound **3** gave the same molecular formula (C_31_H_40_O_12_) as **2** by negative HRESIMS: *m*/*z* 603.2445 [M − H]^−^ (calcd for C_31_H_39_O_12_, 603.2442). Comparison of the NMR data (Table 1) of **3** with those of **2** implied that they were very similar. However, the HMBC correlations of H-2 (*δ*_H_ 4.02, d, *J* = 6.0 Hz) with C-1 (*δ*_C_ 79.0), C-3 (*δ*_C_ 48.7), C-8 (*δ*_C_ 45.6), and C-15 (*δ*_C_ 43.8), and H-14 (*δ*_H_ 5.05, d, *J* = 6.1 Hz) with C-13 (*δ*_C_ 74.3) and C-15 (*δ*_C_ 43.8) revealed that H-2 was shifted upfield from *δ*_H_ 5.65 in **2** to *δ*_H_ 4.02 in **3**, while H-14 was shifted downfield from *δ*_H_ 3.80 in **2** to *δ*_H_ 5.05 in **3**. The above two remarkable shifts (Δ − 1.63 ppm of H-2; Δ + 1.25 ppm of H-14) implied the deesterification and esterification related to aromatic ester, which required a transfer of the benzoyl group from C-2 in **2** to C-14 in **3**. This deduction was further verified by the HMBC correlation of H-14 with the benzoyl carbonyl carbon at *δ*_C_ 169.3. The relative configuration of **3** was established as shown by comparison of relevant coupling constants with those of the isolated analogues. Accordingly, the structure of **3** was determined and named baccatin X.

Compounds **1** and **2** were tested for their cytotoxicity in vitro against five human cancer cell lines: HL-60, SMMC-7721, A-549, MCF-7, and SW480. As summarized in Table 2, **1** was cytotoxic for all the cell lines tested, and displayed moderate activities against HL-60 and MCF-7 (IC_50_ 3.44 and 9.67 μM, respectively), while **2** showed selective cytotoxicity against some of the cell lines.

**Table 2:** Cytotoxicity data for **1** and **2** with IC_50_ values (μM)

Compound	HL-60	SMMC-7721	A-549	MCF-7	SW480
**1**	3.44	14.83	21.27	9.67	22.42
**2**	20.23	16.69	>40	25.32	>40
Cisplatin^a^	1.06	4.32	5.08	15.41	15.35
Taxol^a^	<0.008	<0.008	<0.008	<0.008	<0.008

^a^ Positive controls

## Experimental Section

### General Experimental Procedures

Optical rotations were measured in methanolic solution on a Jasco P-1020 automatic digital polarimeter. IR spectra in potassium bromide discs were taken with a Bruker Tensor 27 FT-IR spectrometer. NMR spectra were performed on Bruker DRX-500 and Avance III 600 instruments with deuterated solvent signals as internal standards. MS data were measured on VG Auto Spec-3000, API QSTAR time-of-flight, and Bruker Esquire HCT spectrometers. Column chromatography was carried out with silica gel (200–300 mesh) and Sephadex LH-20 (Amersham Biosciences, Sweden). Fractions were monitored by TLC and reversed-phase HPLC (Agilent 1200, Agilent Zorbax Extend-C18 column, 5 μm, 4.6 × 150 mm).

### Plant Material

The twigs and leaves of *T*. *yunnanensis* were collected from Yunnan Province of China in February 2008, and identified by Mr. Yu Chen of Kunming Institute of Botany, Chinese Academy of Sciences. A voucher specimen (No. BBP0026016TY) was deposited at BioBioPha Co., Ltd.

### Extraction and Isolation

Dried and powdered twigs and leaves (7.0 kg) of *T. yunnanensis* were extracted with EtOH-H_2_O (95:5, v/v; 3 × 12 L, each 5 days) at room temperature. The combined filtrate was concentrated under vacuum and fractionated by silica gel CC successively eluted with a gradient of increasing acetone in petroleum ether (PE) to obtain four fractions (A–D). Fraction B (PE/Me_2_CO = 6:1) was first chromatographed on silica gel columns, and each major fraction was then purified using a Sephadex LH-20 column (CHCl_3_/MeOH = 1:1) to yield compounds **8** (8 mg), **12** (11 mg), and **13** (45 mg). Fraction C (PE/Me_2_CO = 3:1) was subjected to silica gel CC, eluting with CHCl_3_/MeOH (100:1 → 0:100) to give four fractions (C1–C4). After repeated CC on silica gel (CHCl_3_/Me_2_CO, 10:1 → 0:1) and Sephadex LH-20 (CHCl_3_/MeOH = 1:1), fraction C4 gave compounds **1** (64 mg), **2** (47 mg), **3** (2 mg), **5** (192 mg), **6** (243 mg), and **10** (66 mg). Fraction D (PE/Me_2_CO = 1:1) was purified by repeated CC over silica gel (CHCl_3_/MeOH, 100:1 → 0:100) and recrystallization to afford compounds **4** (327 mg), **7** (226 mg), **9** (884 mg), and **11** (264 mg).

### Baccatin VIII (**1**)

White powder, ${\left[\alpha \right]}_{\text{D}}^{27}$ −15.4 (*c* 0.13, MeOH); UV (MeOH) *λ*_max_: 230, 274 nm; IR (KBr) *ν*_max_: 3433, 2994, 2896, 1726, 1629, 1439, 1373, 1239, 1110, 1054, 1025, 757, 713 cm^−1^; ^1^H and ^13^C NMR data: see Table 1; ESIMS (pos.): *m*/*z* 669 [M + Na]^+^; HRESIMS (pos.): *m*/*z* 669.2513 [M + Na]^+^ (calcd for C_33_H_42_O_13_Na, 669.2523).

### Baccatin IX (**2**)

White powder, ${\left[\alpha \right]}_{\text{D}}^{20}$ +17.3 (*c* 0.18, MeOH); UV (MeOH) *λ*_max_: 230, 274 nm; IR (KBr) *ν*_max_: 3428, 1739, 1719, 1633, 1452, 1435, 1372, 1273, 1168, 1071, 1026, 985, 946, 714 cm^−1^; ^1^H and ^13^C NMR data: see Table 1; ESIMS (pos.): *m*/*z* 627 [M + Na]^+^; HREIMS: *m*/*z* 604.2515 (calcd for C_31_H_40_O_12_, 604.2520).

### Baccatin X (**3**)

White powder; UV (MeOH) *λ*_max_: 230, 273 nm; ^1^H and ^13^C NMR data: see Table 1; ESIMS (pos.): *m*/*z* 627 [M + Na]^+^; HRESIMS (neg.): *m*/*z* 603.2445 [M − H]^−^ (calcd for C_31_H_39_O_12_, 603.2442).

### Cytotoxicity Bioassays

The following human tumor cell lines were used: HL-60, SMMC-7721, A-549, MCF-7, and SW480. All cells were cultured in RPMI-1640 or DMEM medium (Hyclone, Logan, UT, USA), supplemented with 10 % fetal bovine serum (Hyclone) at 37 °C in a humidified atmosphere with 5 % CO_2_. The cytotoxicity assay was performed using the modified MTS (3-(4,5-dimethylthiazol-2-yl)-5-(3-carboxymethoxyphenyl)-2-(4-sulfophenyl)-2*H*-tetrazolium) method as previously described [16]. The IC_50_ value of each compound was calculated by the Reed and Muench’s method [17].

## Electronic supplementary material

Below is the link to the electronic supplementary material.

Supplementary material 1 (DOC 1722 kb)
