# Supplementary material for: New Taxane Diterpenoids from Taxus yunnanensis
Source: Nat Prod Bioprospect. 2014 Feb 19;4(1):47–51. doi: 10.1007/s13659-014-0003-9 (PMC3956972; doi:10.1007/s13659-014-0003-9)
Supplement: Supplementary file 1 — Supplementary material 1 (DOC 1722 kb) [file 13659_2014_3_MOESM1_ESM.doc]

**New taxane diterpenoids from *Taxus yunnanensis***

Ping HAI,^a^ Shi-Zhen WEN,^a^ Yan LI,^b^ Yuan GAO,^a,b^ Xian-Jun JIANG,^a^ and Fei WANG^a,b^,*

^a^BioBioPha Co., Ltd., Kunming 650201, China

^b^State Key Laboratory of Phytochemistry and Plant Resources in West China, Kunming Institute of Botany, Chinese Academy of Sciences, Kunming 650201, China

*To whom correspondence should be addressed. E-mail: [wangfei@mail.kib.ac.cn](mailto:wangfei@mail.kib.ac.cn) (F. Wang)

Structures of compounds **1**–**3**

**Content list:**

**S1.** ^1^H NMR spectrum (500 MHz, CD_3_OD) of baccatin VIII (**1**).

**S2.** ^13^C NMR (DEPT) spectrum (100 MHz, CD_3_OD) of baccatin VIII (**1**).

**S3.** HMBC spectrum (500 MHz, CD_3_OD) of baccatin VIII (**1**).

**S4.** HSQC spectrum (500 MHz, CD_3_OD) of baccatin VIII (**1**).

**S5.** ROESY spectrum (500 MHz, CD_3_OD) of baccatin VIII (**1**).

**S6.**  ^1^H NMR spectrum (500MHz, DMSO-*d*_6_) of baccatin IX (**2**).

**S7.**  ^1^H NMR spectrum (400MHz, CD_3_OD) of baccatin IX (**2**).

**S8.** ^13^C NMR (DEPT) spectrum (100 MHz, DMSO-*d*_6_) of baccatin IX (**2**).

**S9.**  HMBC spectrum (500 MHz, DMSO-*d*_6_) of baccatin IX (**2**).

**S10.** ROESY spectrum (500 MHz, DMSO-*d*_6_) of baccatin IX (**2**).

**S11.** ^1^H NMR spectrum (600MHz, CD_3_OD) of baccatin X (**3**).

**S12.** ^13^C NMR (DEPT) spectrum (150 MHz, CD_3_OD) of baccatin X (**3**).

**S13.** HMBC spectrum (600 MHz, CD_3_OD) of baccatin X (**3**).

**S14.** ESIMS spectrum of baccatin VIII (**1**).

**S15.** HRESIMS spectrum of baccatin VIII (**1**).

**S16.** ESIMS spectrum of baccatin IX (**2**).

**S17.** HREIMS spectrum of baccatin IX (**2**).

**S18.** ESIMS spectrum of baccatin X (**3**).

**S19.** HRESIMS spectrum of baccatin X (**3**).

**S20.** HPLC analysis and its UV spectrum of baccatin VIII (**1**).

**S21.** HPLC analysis and its UV spectrum of baccatin IX (**2**).

**S22.** HPLC analysis and its UV spectrum of baccatin X (**3**).

**S1.** ^1^H NMR spectrum (500 MHz, CD_3_OD) of baccatin VIII (**1**).


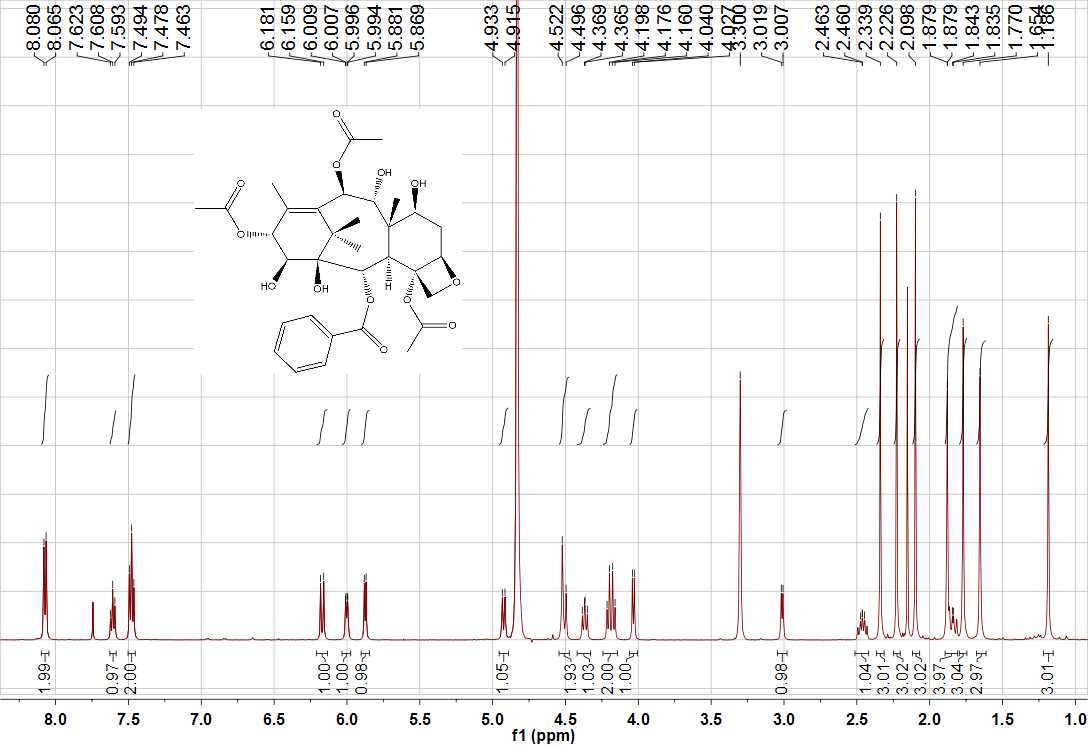


**S2.** ^13^C NMR (DEPT) spectrum (100 MHz, CD_3_OD) of baccatin VIII (**1**).


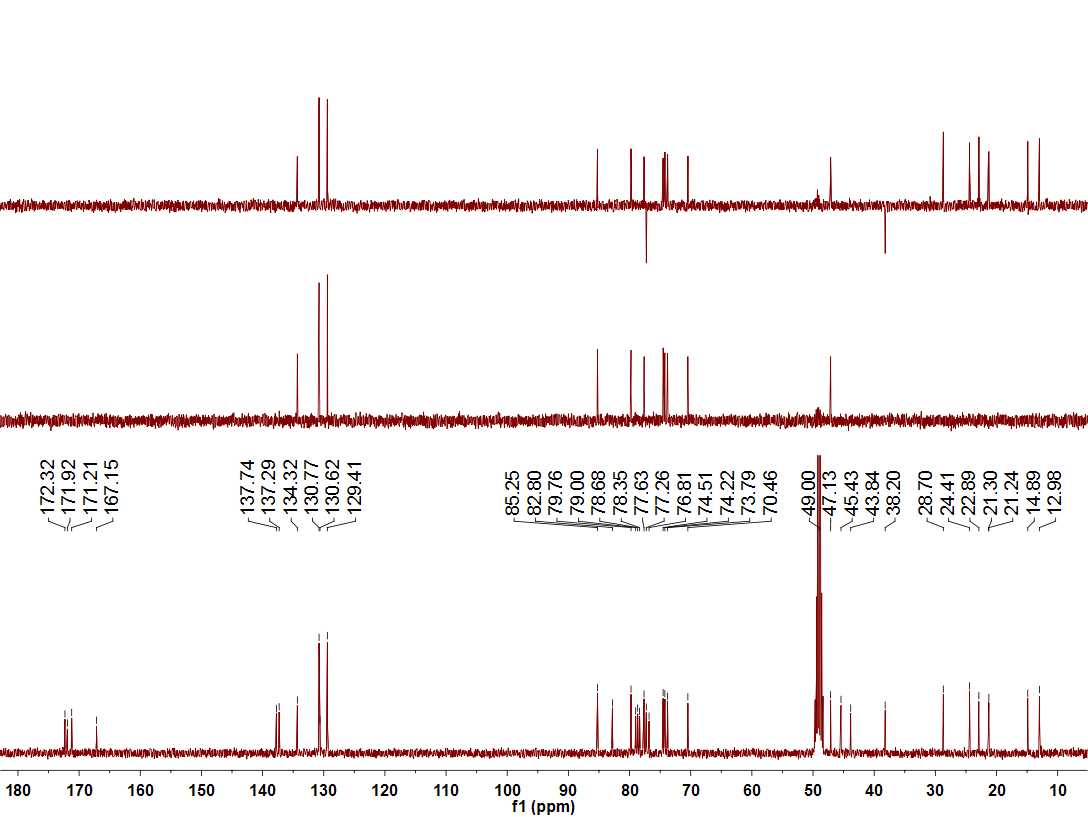


**S3.** HMBC spectrum (500 MHz, CD_3_OD) of baccatin VIII (**1**).

**
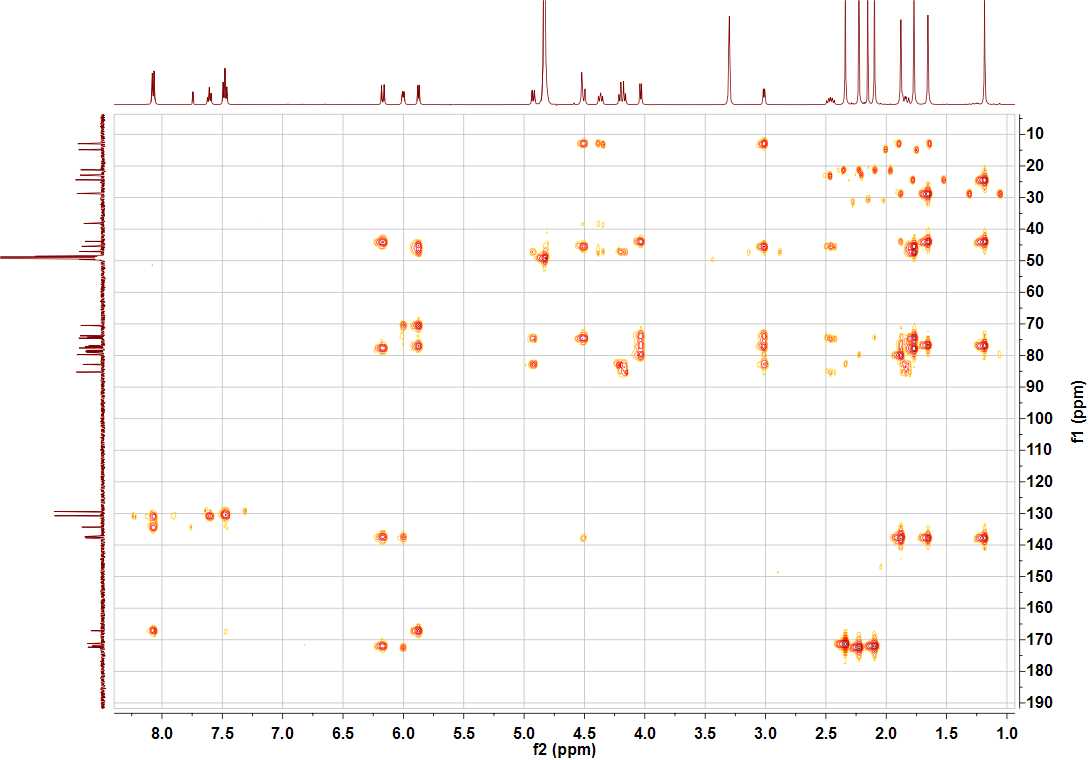
**

**S4.** HSQC spectrum (500 MHz, CD_3_OD) of baccatin VIII (**1**).


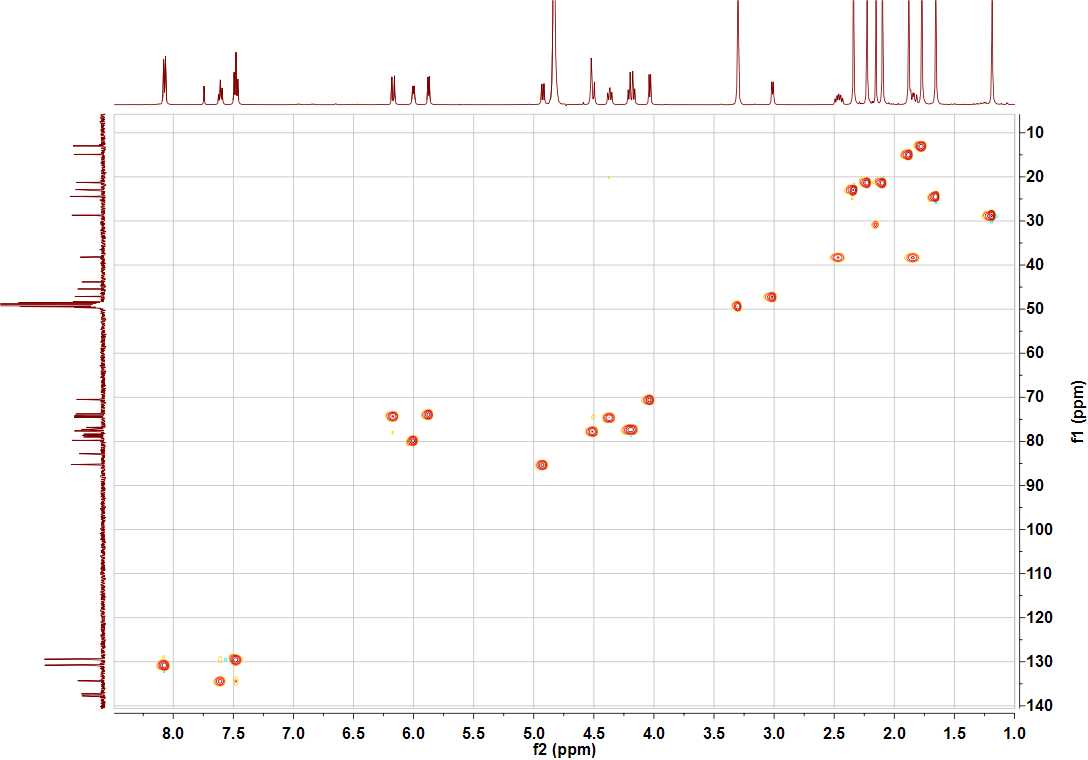


**S5.** ROESY spectrum (500 MHz, CD_3_OD) of baccatin VIII (**1**).


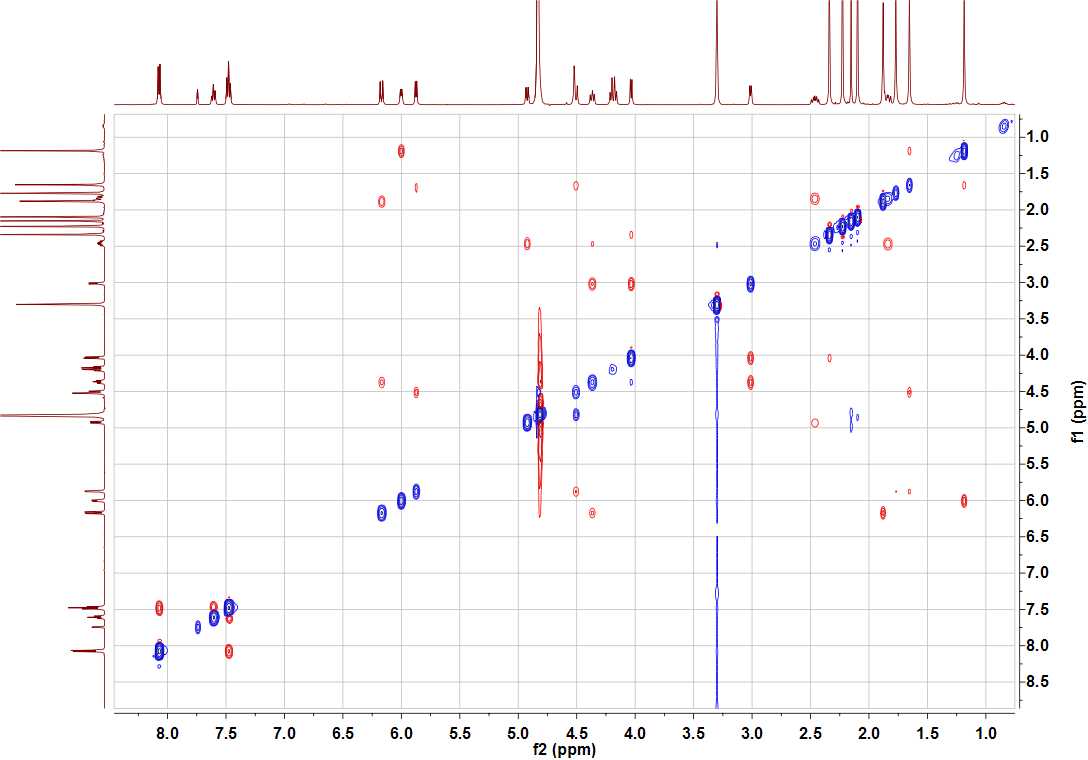


**S6.** ^1^H NMR spectrum (500MHz, DMSO-*d*_6_) of baccatin IX (**2**).


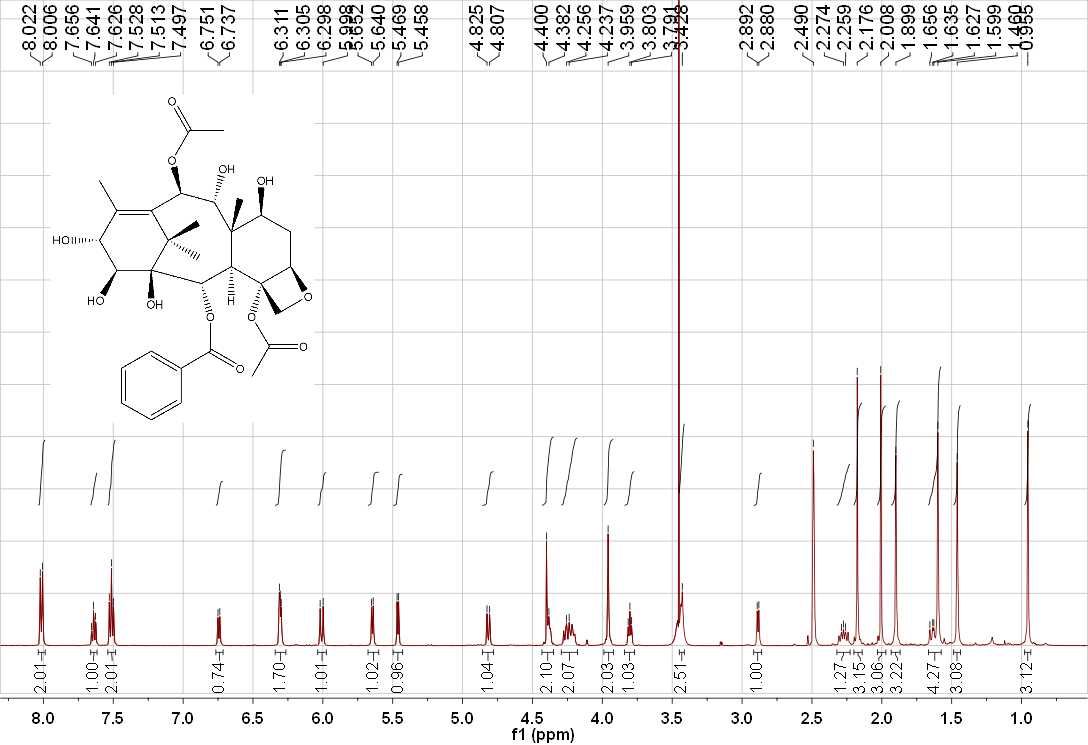


**S7.** ^1^H NMR spectrum (400MHz, CD_3_OD) of baccatin IX (**2**).


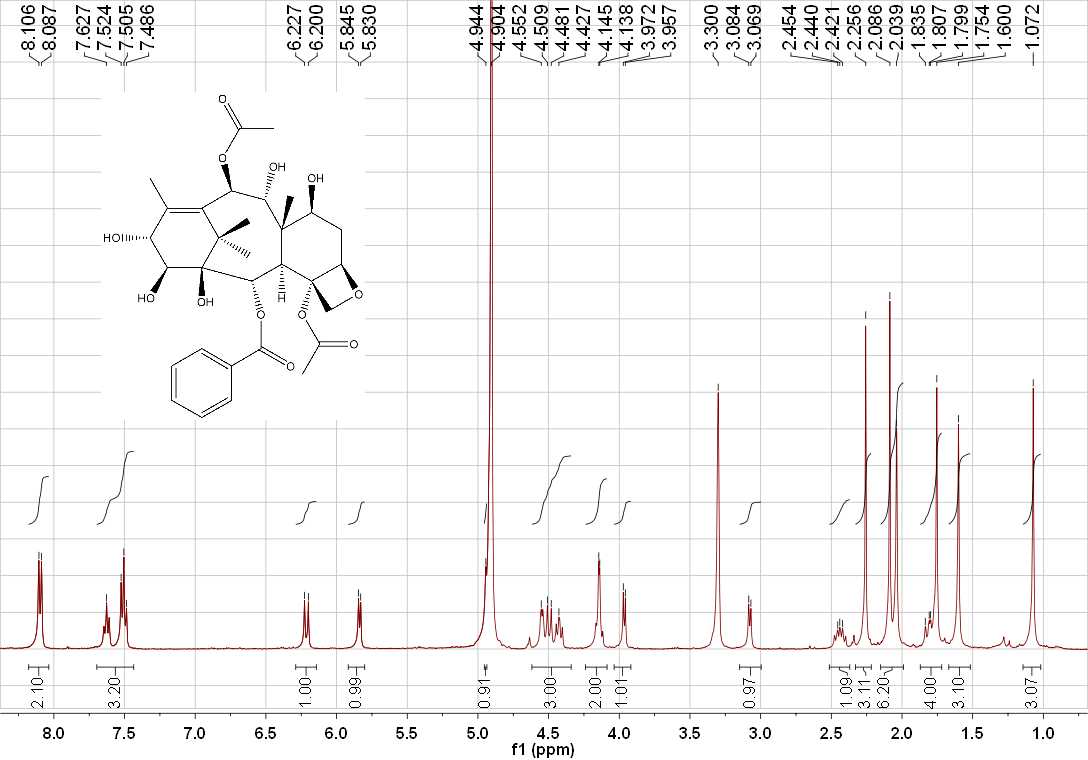


**S8.** ^13^C NMR (DEPT) spectrum (100 MHz, DMSO-*d*_6_) of baccatin IX (**2**).


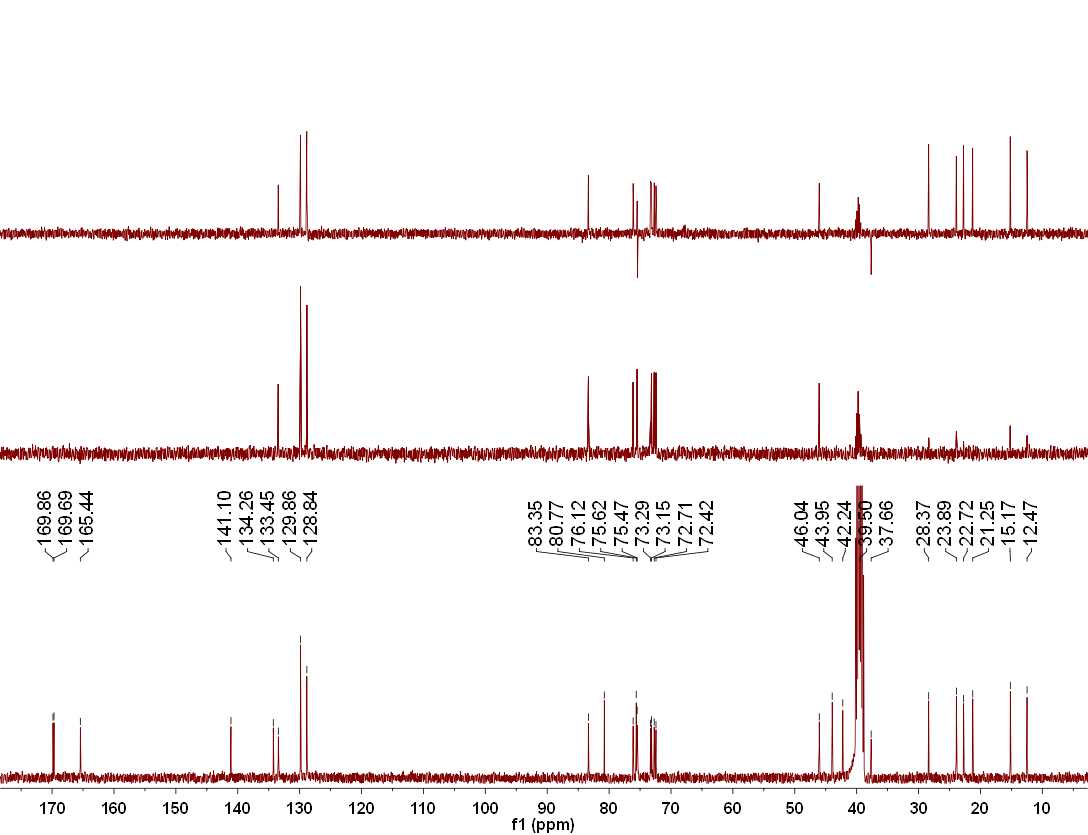


**S9.** HMBC spectrum (500 MHz, DMSO-*d*_6_) of baccatin IX (**2**).

**
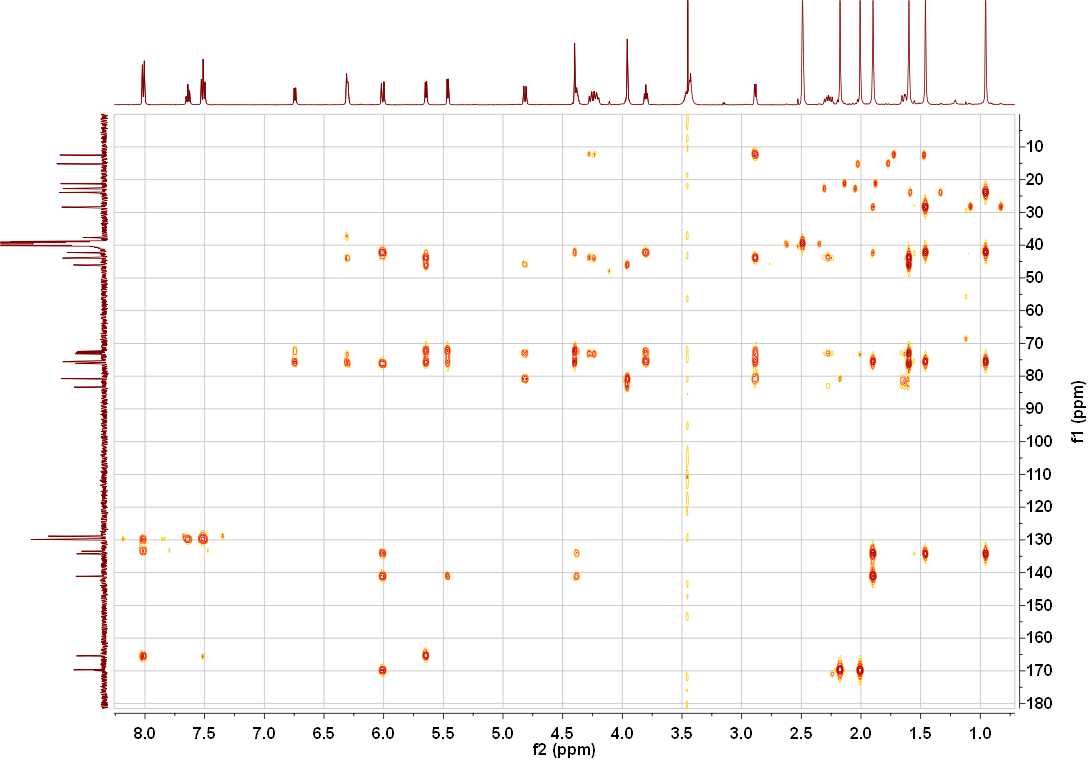
**

**S10.** ROESY spectrum (500 MHz, DMSO-*d*_6_) of baccatin IX (**2**).

**
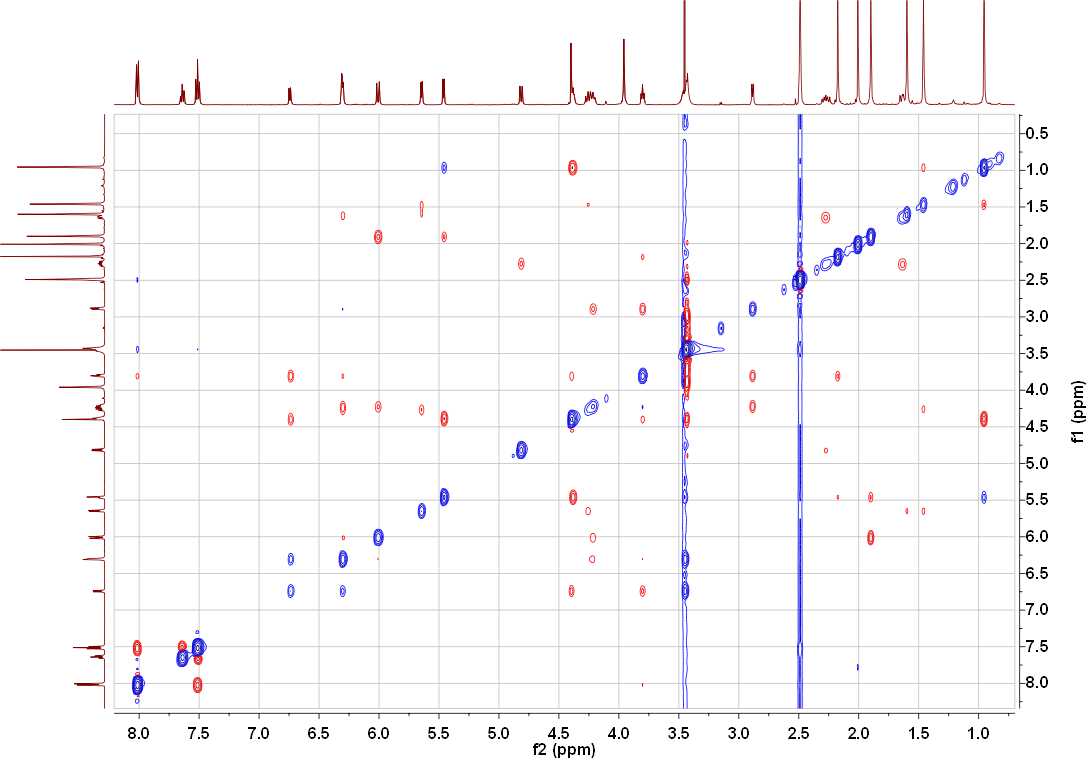
**

**S11.** ^1^H NMR spectrum (600MHz, CD_3_OD) of baccatin X (**3**).

**
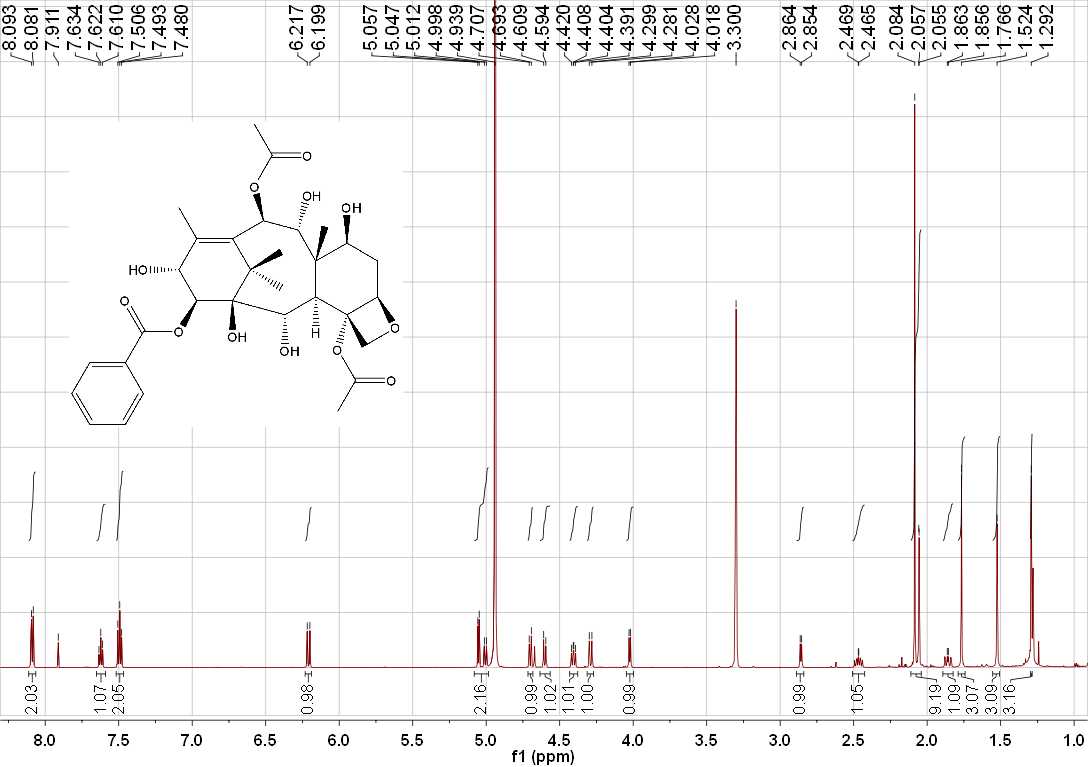
**

**S12.** ^13^C NMR (DEPT) spectrum (150 MHz, CD_3_OD) of baccatin X (**3**).

**
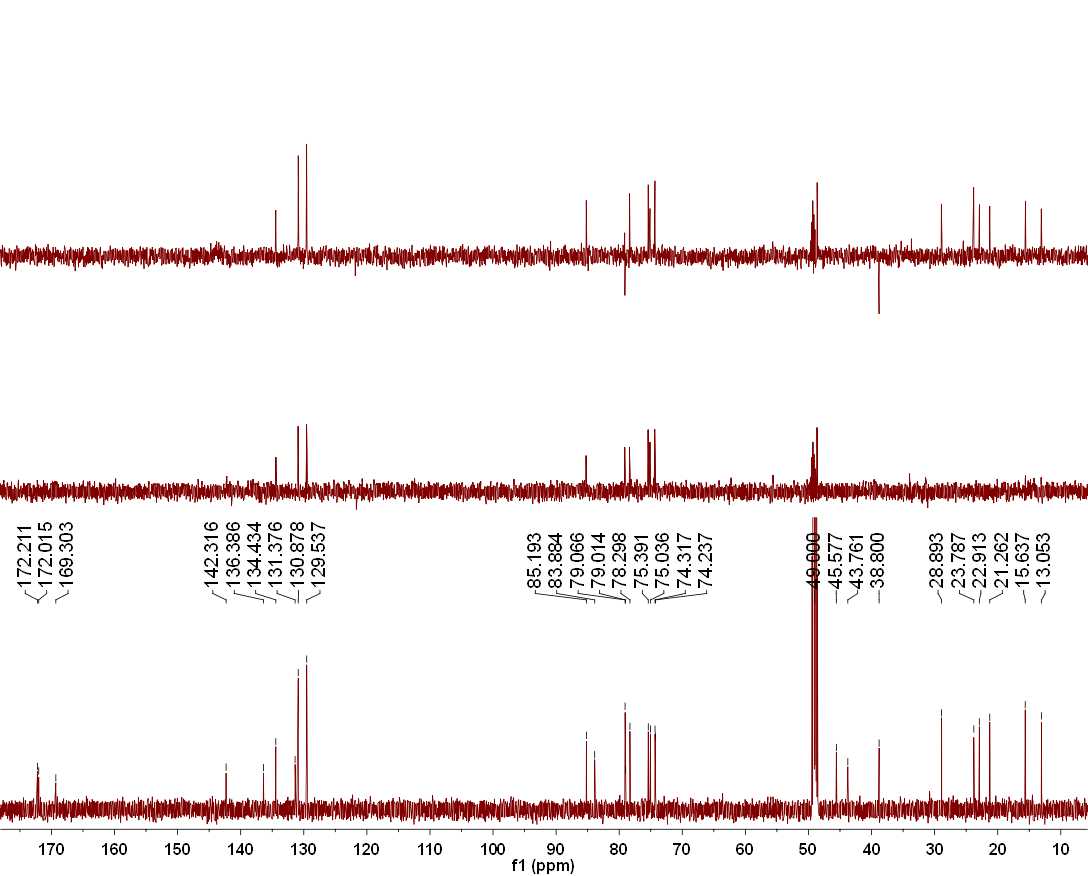
**

**S13.** HMBC spectrum (600 MHz, CD_3_OD) of baccatin X (**3**).


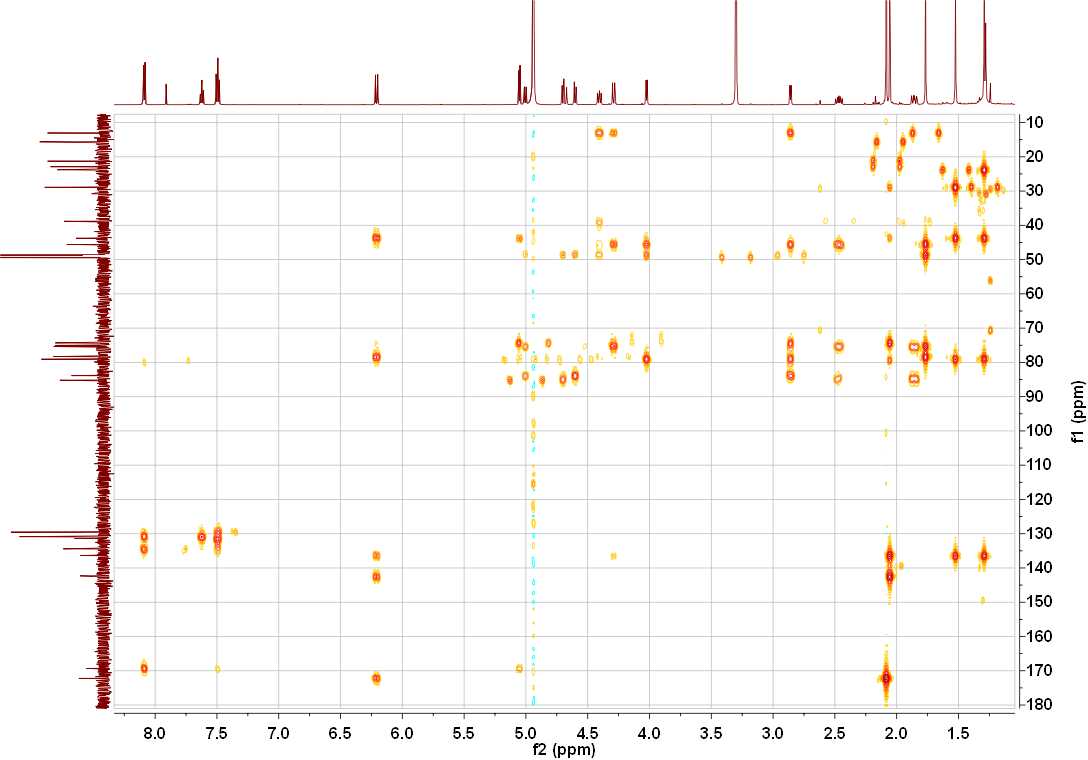


**S14.** ESIMS spectrum of baccatin VIII (**1**).

**
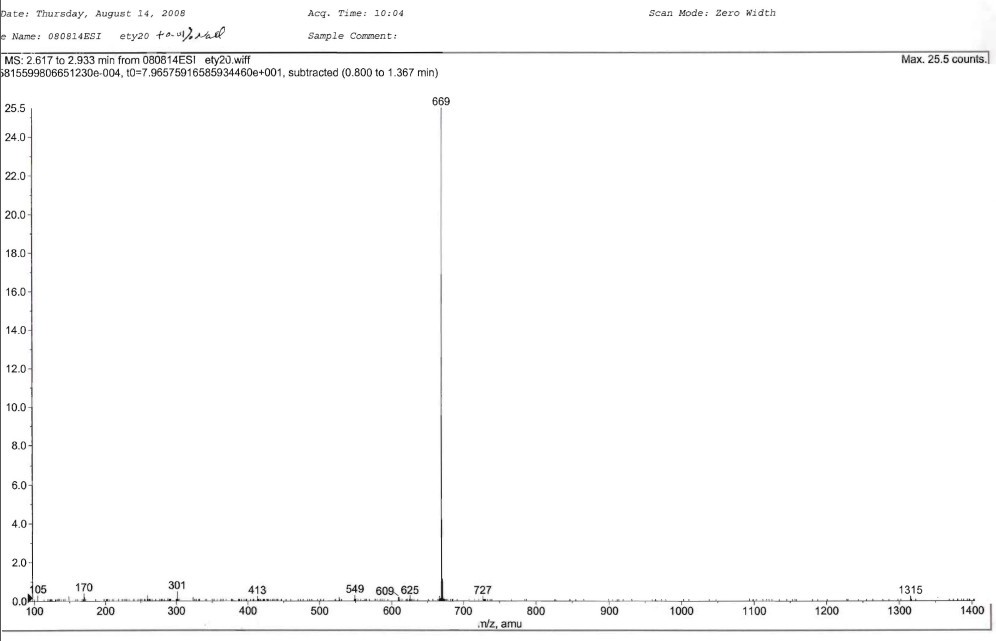
**

**S15.** HRESIMS spectrum of baccatin VIII (**1**).


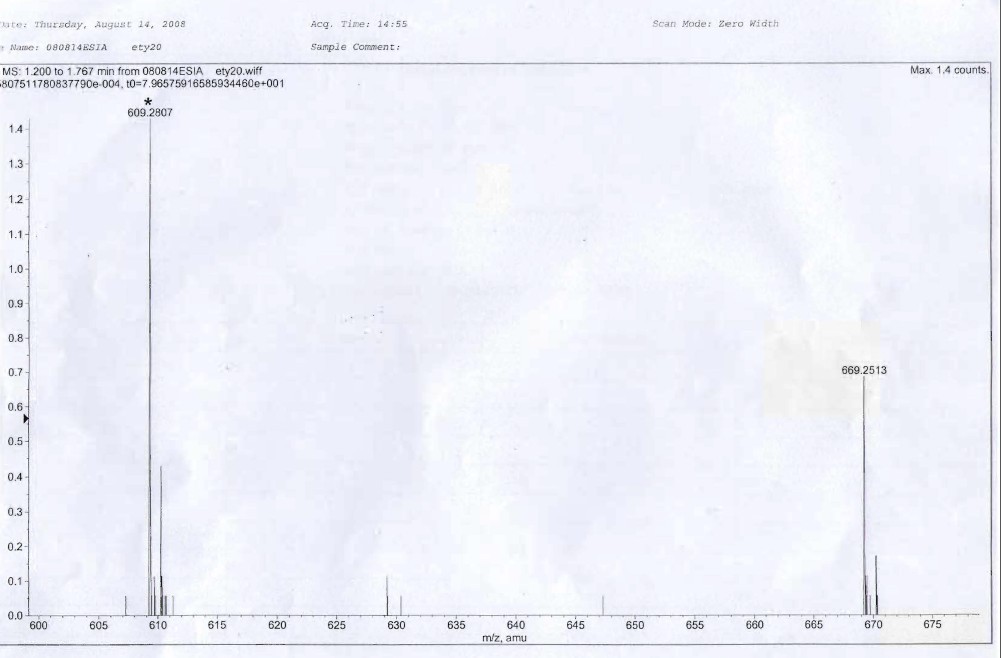


**
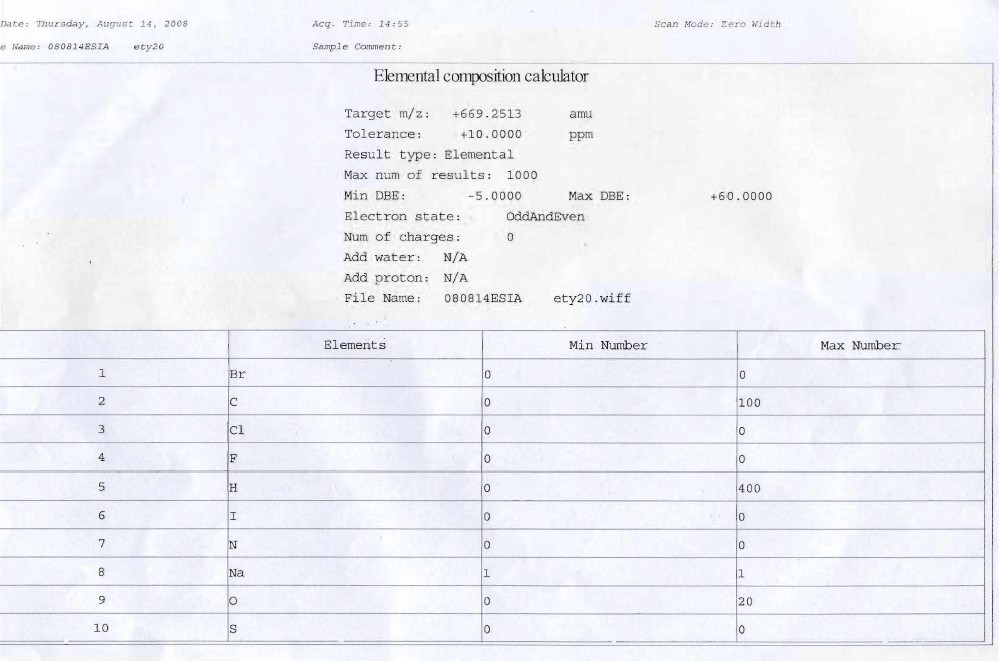
**

**
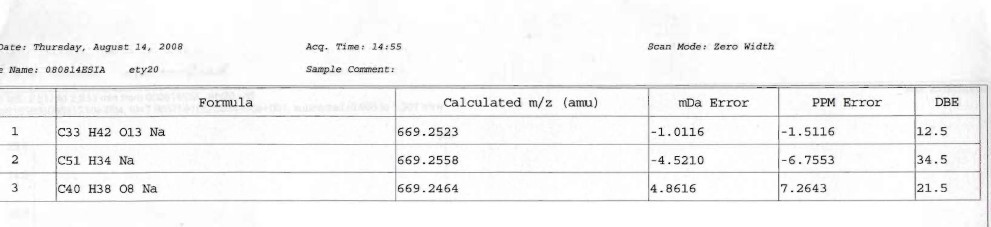
**

**S16.** ESIMS spectrum of baccatin IX (**2**).

**
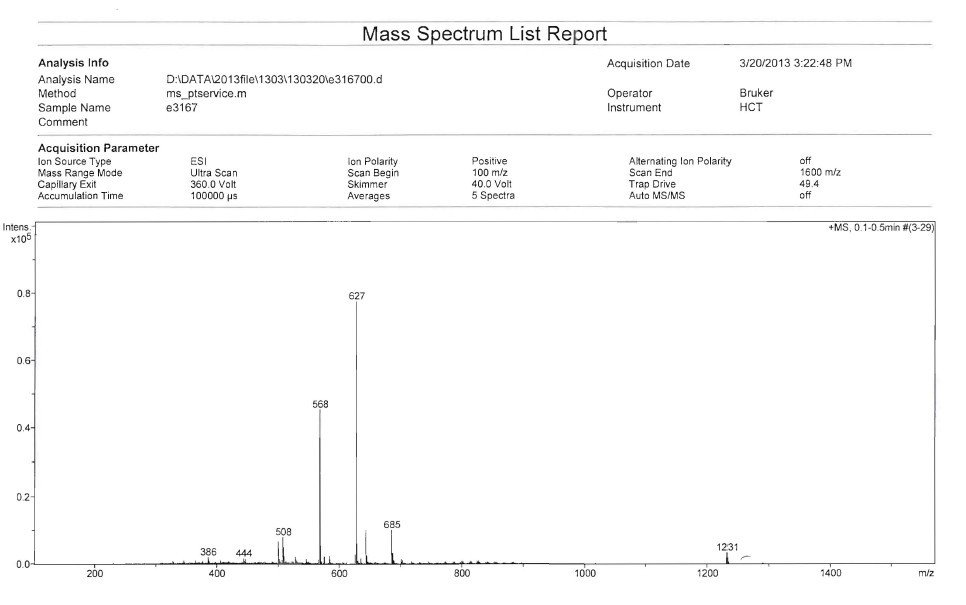
**

**S17.** HREIMS spectrum of baccatin IX (**2**).

**
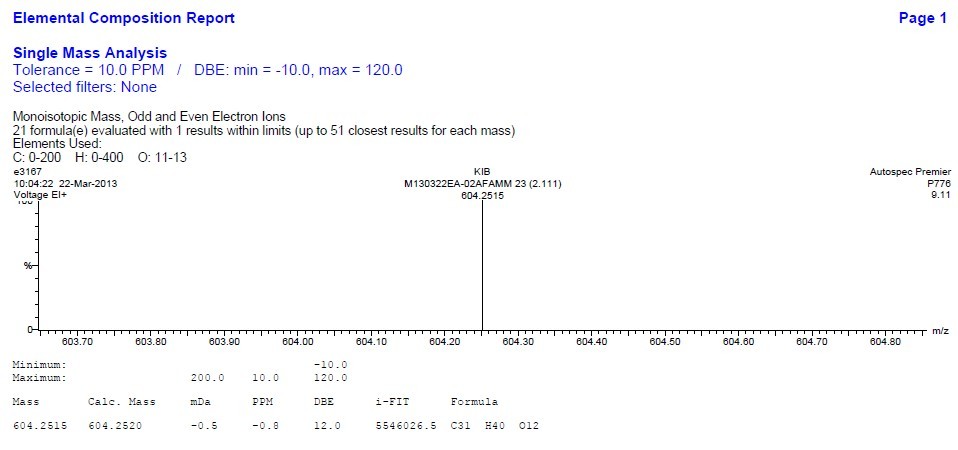
**

**S18.** ESIMS spectrum of baccatin X (**3**).

**
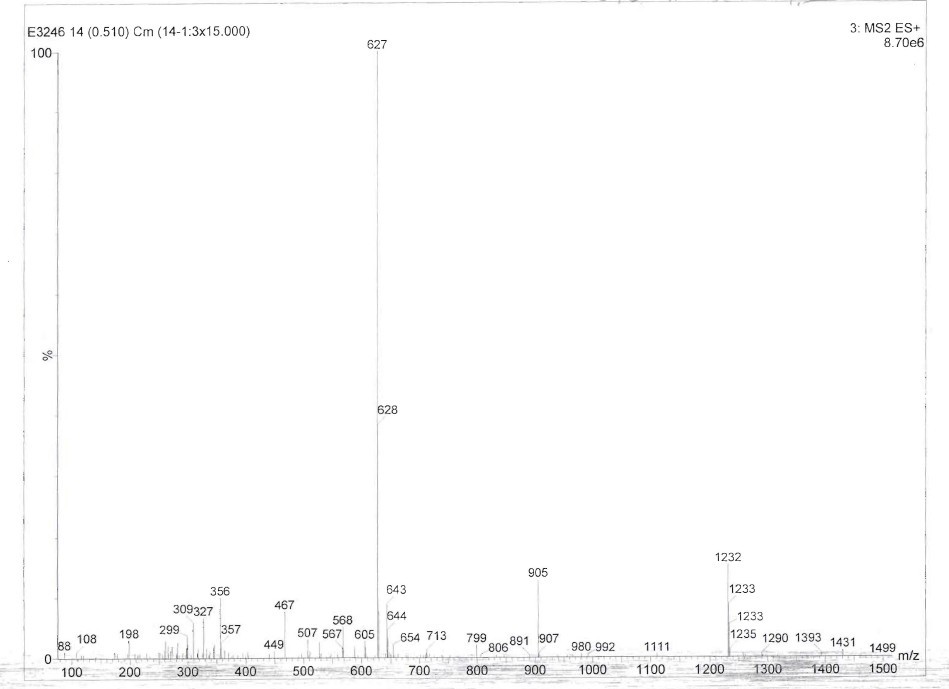
**

**S19.** HRESIMS spectrum of baccatin X (**3**).

**
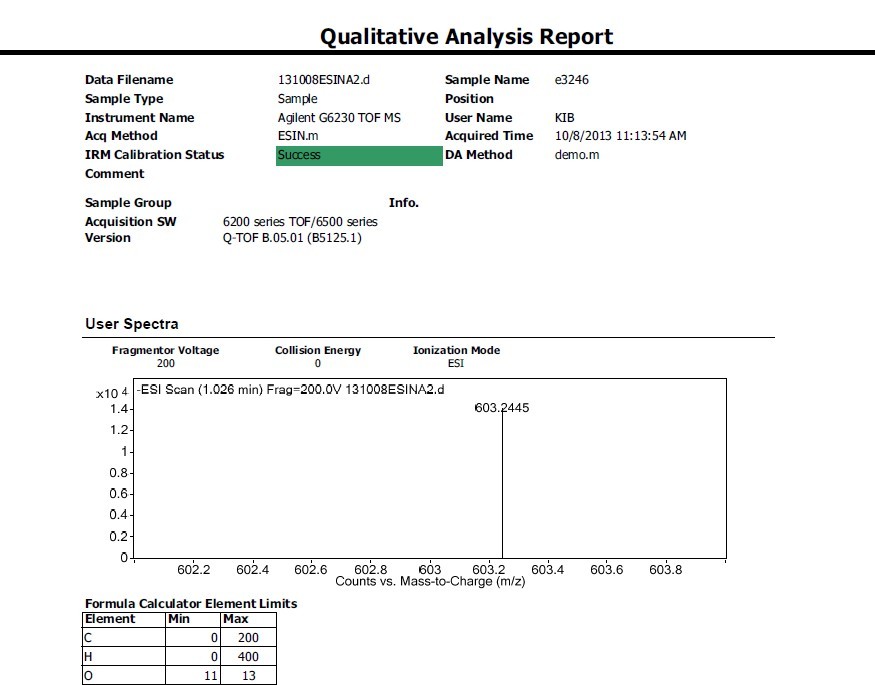
**

**
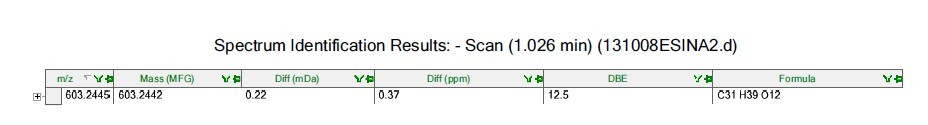
**

**S20.** HPLC analysis and its UV spectrum of baccatin VIII (**1**).

**S21.** HPLC analysis and its UV spectrum of baccatin IX (**2**).

**S22.** HPLC analysis and its UV spectrum of baccatin X (**3**).
